# The effects of spatial relations and motion information in social scene perception

**DOI:** 10.1038/s41598-025-07870-1

**Published:** 2025-07-16

**Authors:** Violette Munin, Etienne Abassi, Pierre-Aurélien Beuriat, Liuba Papeo

**Affiliations:** 1 Institute of Cognitive Sciences Marc Jeannerod, UMR 5229, CNRS and Université Claude Bernard Lyon 1, 67 Boulevard Pinel, 69500 Bron, France; 2 Department of Pediatric Neurosurgery, Hospices Civils de Lyon, Hôpital Femme Mère Enfant, Bron, France; 3 Université Claude Bernard Lyon 1, Villeurbanne, France

**Keywords:** Social cognition, Social perception, Motion perception, Brain lateralization, fMRI, Perception, Social neuroscience, Visual system

## Abstract

A body of research shows that the human visual system hosts a network specialized in processing social interactions—i.e., physical/communicative exchanges between people. This network largely overlaps with regions involved in motion perception. Current views propose that, since motion is an intrinsic component of social interaction, it is necessary to trigger social-interaction selectivity. However, the relationship between social-interaction perception and motion perception remains unclear. We took advantage of two existing functional MRI datasets collected in the same participants, to identify the social-interaction network, and study how it responds to static versus dynamic visual social scenes. Results showed that scenes showing interacting face-to-face (vs. back-to-back) people elicited greater activity in the extrastriate body area (EBA) and posterior superior temporal sulcus (pSTS). This *facingness effect* was observed for both static and dynamic stimuli, indicating that motion is not required to trigger social-interaction selectivity. However, neural activity was stronger for dynamic (vs. static) stimuli, implying that motion enhances the network’s responsiveness. Moreover, the *facingness effect* was stronger in left hemisphere areas. Thus, while social perception has traditionally been associated with the right hemisphere**,** the present findings highlight a critical role for left visual areas, raising new questions about their function in social perception.

**Supplementary Information:**

The online version contains supplementary material available at 10.1038/s41598-025-07870-1.

## Introduction

A social interaction is an event in which at least one agent acts intentionally to affect the state of another agent. The scale, diversity and complexity of human social interactions are unmatched in the animal kingdom. In latest years, cognitive neuroscience research has highlighted specialized mechanisms and brain areas for detecting and processing social interactions in the visual world^1–7^. Observing two people looking at, or moving toward each other (i.e., face-to-face or *facing*) recruits particularly efficient visual perception mechanisms^5,8–10^. In functional MRI (fMRI) studies, visual perception of social interactions has been associated with increased activity in lateral visual areas, overlapping with or adjacent to areas involved in perception of bodies and bodily motion, in the extrastriate body area (EBA) and posterior superior temporal sulcus (pSTS)^1–4,11–17^.

The replication of the effect across different studies, stimulus-sets and tasks (see Table 1) has made the increased neural response to facing/interacting versus non-facing/non-interacting bodies a reliable signature of social interaction selectivity. This univariate effect with visually matched stimuli (most often, the very same bodies and body movements were presented in the interacting and non-interacting contexts) may reflect additional integrative processing and emerging properties of related/interacting individuals^18–20^. Overall, this body of studies converges on a role of the visual system in the earliest stages of social interaction processing: a network of visual areas would leverage relational cues in the stimulus structure (e.g., spatial positioning of bodies, body postures, distance) to begin the transformative process that goes from body and motion perception to representation of social interaction^3,14^.

**Table 1:** Papers (ordered by stimulus modality, regions activated and lateralization of the activations) documenting social interaction selectivity in lateral visual areas, using static or dynamic representations of minimal social scenes (i.e., facing vs. non-facing people) or fully-fledged, meaningful interactions.

Stimuli		Brain regions				Notes
Meaningful interactions	Minimal social scenes	EBA	pSTS/STG	MT	Other visual areas	
	**Abassi and Papeo** ^1^	**L**			**R-MOG**	
	**Abassi and Papeo** ^2^	**L**				
	**Abassi and Papeo** ^11^	**L**			**FBA**	
	**Gandolfo et al.** ^21^	**L**				
**Walter et al.** ^22^			**Bilateral**		**Bilateral MTG**	
**Kujala et al.** ^23^			**Bilateral**		**R-MTG, L-FG**	
**Quadflieg et al.** ^24^						**No effect for interacting > non-interacting dyads**
**Pierno et al.** ^25^					**Bilateral MOG**	
*Atkinson and Vuong* ^26^					*R-MTG, R-aSTS*	
*Schultz et al.* ^27^			*R*		*Bilateral MOG*	
*Masson and Isik* ^28^			*R*			
*Isik et al.* ^4^			*R*			
*Walbrin et al.* ^7^			*R*		*R-LOTC*	*Only right ROIs were tested*
*Iacoboni et al.* ^29^			*R*	*Bilateral*	*R-FG*	
*Castelli et al.* ^30^			*Bilateral*		*Bilateral Occipital lobe*	
*Centelles et al.* ^31^			*Bilateral*		*R-MTG, R-FG*	
*Georgescu et al.* ^32^			*Bilateral*		*Bilateral MTG* *Bilateral FG*	
*Lahnakoski et al.* ^33^			*Bilateral*		*Bilateral ITC*	
*Masson et al.* ^34^			*Bilateral*		*L-IOG/MTG, R-MTG*	*IOG/MTG coordinates are compatible with EBA*
*Santos et al.* ^35^			*Bilateral*			
*Sapey-Triomphe et al.* ^36^			*Bilateral*		*R-MOG*	
*Schultz et al.* ^21^			*Bilateral*			
*Wurm et al.* ^17^			*Bilateral*		*Bilateral LOTC/MTG*	
*Wurm and Caramazza* ^16^			*Bilateral*		*Bilateral LOTC*	
*Walbrin et al.* ^37^			*Bilateral*			*Marginal selectivity in left EBA (p* = *0.054)*
*Dolcos et al.* ^38^		*R*	*Bilateral*		*L-MTG*	*From whole brain contrast [approach* + *avoid* > *control]*
	*Bellot et al.* ^3^	*Bilateral*	*Bilateral*			
*Walbrin and Koldewyn* ^15^		*Bilateral*	*Bilateral*			
*Masson et al.* ^13^		*Bilateral*	*Bilateral*			
***Landseidel et al. (2022)*** 12		***Bilateral***	***Bilateral***	***R***	***L-FBA***	

Bold = static stimuli, italic = dynamic stimuli, bolditalic = static and dynamic stimuli; *L* left activity, *Right* right activity; MOG = middle occipital gyrus; FBA = fusiform body area; MTG = middle temporal gyrus; FG = fusiform gyrus; LOTC = lateral occipital temporal cortex; ITC = inferior temporal cortex; IOG = inferior occipital gyrus;

The functional specificity of the areas within this network is currently under investigation. An outstanding question concerns the role of motion. Current views propose that motion is an intrinsic component of social interaction and, therefore, it is necessary to trigger social-interaction selectivity^12,39–41^. This view is supported by the fact that social-interaction selectivity in pSTS was reported in studies using dynamic stimuli^21,27,28^ (see also Table 1), but not in most studies using static stimuli^1,2,11,20,25,31^. Other findings however reported an effect in pSTS also for static stimuli^22,23^. Moreover, while some studies reported an effect in EBA for both static^1,2,11,20^ and dynamic stimuli^3,13,15,38^, other studies did not find EBA activity using either static^22–25^ or dynamic stimuli^29,30,32–37^ (see also Table 1).

One problem with this inconsistent set of results is that the available studies used either static or dynamic stimuli, making it difficult to compare effects between stimulus modalities, and to conclude on whether, and why, a given region would respond more (or selectively) to one or the other modality.

An exception is the work of Landsiedel and colleagues^12^, who measured neural activity during perception of both video-clips and static frames of interacting versus non-interacting dyads, and showed social-interaction selectivity in pSTS and EBA in the dynamic (video-clip) condition only. However, as the authors noted, there are at least two ways in which ‘social interaction’ has been operationalized in the current literature. Some studies, including Landsiedel et al.^12^, used representations of meaningful, fully-fledged dyadic social interactions (compared with individuals acting in isolation), where the interaction was indicated by a variety of cues such as action categories and their coherence, object- and scene-level properties (which objects are involved and where the event takes place) and other contextual cues (e.g., clothing), emotional cues, in addition to merely physical properties, such as distance, motion direction, and/or body orientation^14^. Other studies instead selected and systematically varied only key ‘prototypical’ physical cues of social interaction such as distance and body orientation, under the hypothesis that the visual system is tuned for quick and accurate perception of nearby face-to-face (vs. non-facing) bodies^5,6^. On this view, the *facing* configuration of two bodies would constitute the most basic structure that the visual system readily *reads* as ‘social interaction’. Thus, the question is: If a visual scene features just two people close together and face-to-face, is motion still necessary to elicit interaction selectivity in EBA and pSTS? This question comes closer to understanding what ‘social interaction’ is for the human visual system.

A way to address this question is to measure neural responses to static and dynamic stimuli that are comparable in terms of physical structure, with just two nearby bodies oriented toward (vs. away from) each other. In this study, we did so, with opportunistic analyses on two existing fMRI datasets measuring neural activity in response to, respectively, static and dynamic body dyads that did not depict any familiar, easy to identify, social interaction, and carry no interaction cues other than *facingness*. To favor comparison between stimulus sets, our analyses only considered fMRI data from participants who took part in both studies (i.e., with static and dynamic stimuli).

To preview, we found that facing dyads—both static and dynamic—triggered left-lateralized effects in both the lateral occipital cortex overlapping with the EBA, and the pSTS. This effect demonstrates that motion is not a necessary signal to trigger social interaction selectivity in the ‘social interaction’ visual-perception network. Furthermore, left-lateralized effects challenge the common view of a right-hemisphere superiority in social perception, opening new questions about the function of left brain areas in social perception and cognition.

## Methods

### Participants

Fifteen healthy adults participated in two distinct fMRI sessions as paid volunteers (7 identified themselves as female; 8 identified themselves as male; mean age: 25.2 ± 4.6 *SD*). They were part of two larger groups involved in two published studies, one involving static stimuli^2^, and the other involving dynamic stimuli^3^. All participants had normal or corrected-to-normal vision. They reported no history of medical, psychiatric, or neurological disorders, or use of psychoactive medications. They were screened for contraindications to fMRI and gave informed consent before participation. A sensitivity analysis was conducted using G*Power^42^ to determine the minimum detectable effect size given the study parameters. Assuming a within-subjects design with 40 conditions, a sample size of N = 15, an alpha level of 0.05, and a desired power of 0.80, the analysis indicated a minimum detectable effect size of f = 0.185. Thus, the study was adequately powered to detect effects in the small-to-medium range. Experimental procedures were approved by the local ethics committee and the study was conducted in accordance with the relevant guidelines and regulations. Data collection was carried out at the CERMEP neuroimaging center in Bron, after approval of the ethics committee (Comité de Protection des Personnes—CPP Sud Est V, Centre Hospitalier Universitaire—CHU de Grenoble).

### Stimuli

#### Static stimuli

**Fig. 1:**
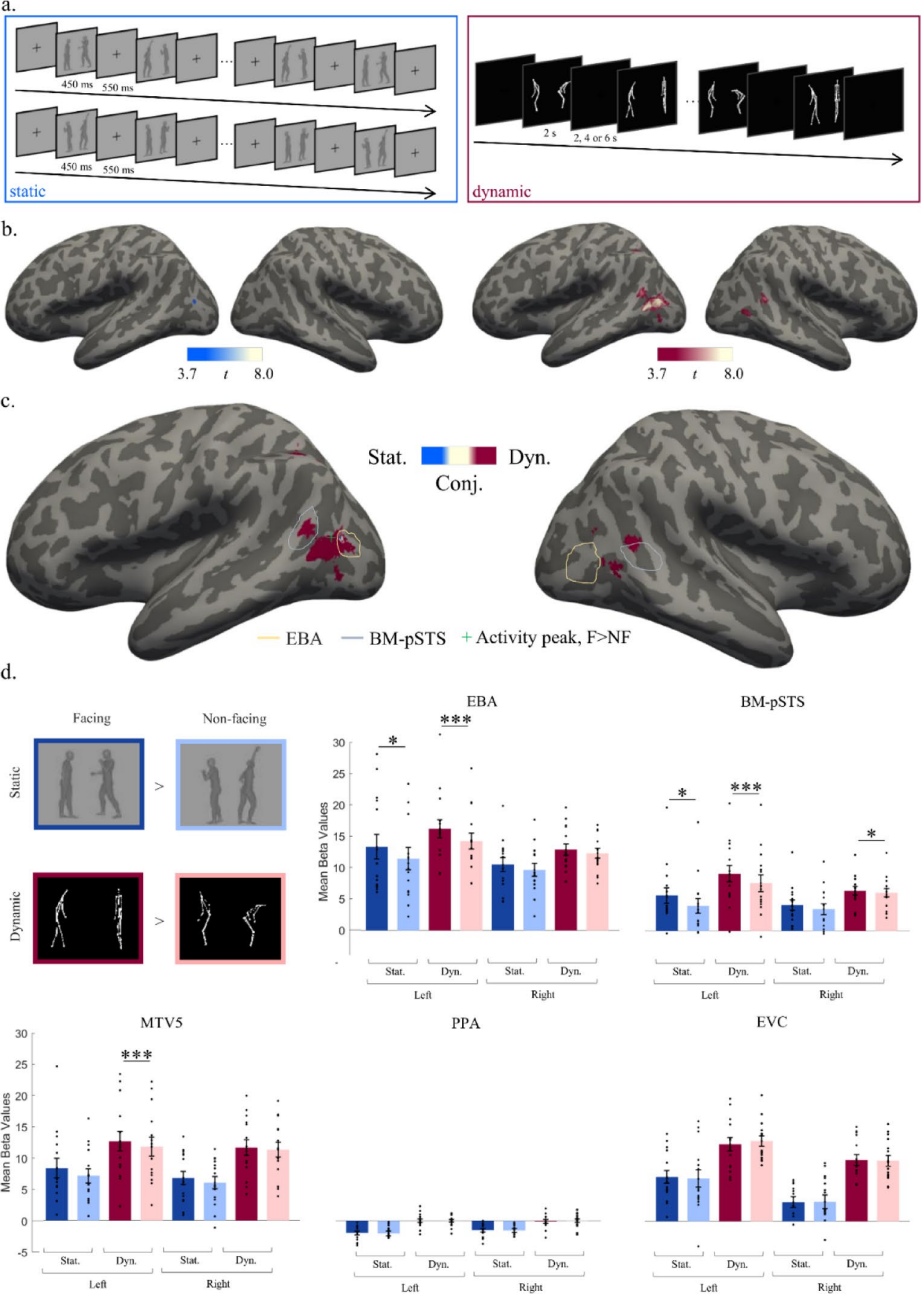
(**a**) fMRI design for the sessions with static (blue) and the dynamic (red) stimuli. Lines connecting dots in point-light displays were added for visualization purposes only; (**b**) Results of the whole brain contrast facing > non-facing for static (left) and dynamic (right) stimuli; *p* = 0.001, FDR-corrected; (**c**) Results of the conjunction analysis of group-level maps for the contrast facing > non-facing for static (blue) and dynamic (red) stimuli; *p* = 0.001, FDR-corrected. The conjunction is highlighted in beige. The functionally-defined group-level EBA is highlighted in yellow. The functionally-defined group-level BM-pSTS is highlighted in blue. Activity peak is plotted with a green cross; (**d**) Region of interest (ROI) analysis in EBA, BM-pSTS, MTV5, PPA and EVC. Error bars denote the within-subjects normalized SEM. ∗*p* ≤ 0.05; ∗∗*p* ≤ 0.01; ∗∗∗*p* ≤ 0.001. Black dots represent each single subject’s mean activity across the ROI. Bars represent mean beta values for facing and non-facing activity across all subjects. Only significance of the configuration effect is plotted. Details of other main effects and interaction can be found in the results section.

Facing dyads were formed from 16 gray-scale renderings of human bodies (and their mirror version), all in profile view and biomechanically possible poses, for a total of 32 bodies. Single bodies were randomly paired to form 16 facing dyads, and their mirror version, for a total of 32 facing dyads (Fig. 1a). Bodies in each dyad were horizontally flipped to form 32 non-facing dyads. The centers of the two bounding boxes that contained the two bodies in a dyad were equally distant from the center of the image. The distance between two bodies in a dyad was matched across facing and non-facing dyads (mean_facing_ = 82.88 pixels ± 13.76 *SD*; mean_non-facing_ = 83.06 ± 13.86; *t*(15) = 1.00; *p* > 0.250). In sum, facing and non-facing stimuli were identical except for the relative positioning of the two bodies.

#### Dynamic stimuli

Dynamic stimuli (Fig. 1a) consisted of silent movies of 2000 ms, showing point-light displays (PLDs) of two human bodies performing two different movements. PLDs depict the movements of two bodies by means of few isolated points in correspondence with the major joints of the moving body, thus allowing to isolate and closely control body motion (i.e., kinematic and shape) information^43^. The movement of each body was depicted by 13 white dots (on a black background) in correspondence with the major joints of the moving body (top of the head, shoulders, elbows, hands, hips, knees, and ankles). The 20 individual PLD-bodies that formed the dyads were selected from a public database^44^ and randomly paired, yielding 10 facing and 10 non-facing dyads, without any obvious, familiar content. Bodies were oriented toward or away from each other. The distance between the two bodies as well as the average motion energy (i.e., optical flow^45^) was matched between facing and non-facing dyads (distance: mean_facing_ = 85 pixels ± 82 *SD*; mean_non-facing_ = 92 ± 79; t(9) < 1, *ns;* optical flow: mean magnitude_facing_ + − sd = 2.19 + − 0.60, mean magnitude_non-facing_ = 2.21 ± 0.60 *SD*, *t*(9) = 1.26, *p* = 0.24). More details on the stimuli can be found in Abassi and Papeo^2^ and Bellot et al.^3^, respectively.

### Procedures

Static and dynamic stimuli were presented to participants in two different sessions, performed on different days with an interval of about 1 month between the two sessions (Fig. 1a). The order of the sessions was counterbalanced across participants. The session with static stimuli included blocks of single bodies, single objects, facing and non-facing dyads, all presented upright and inverted (in separate blocks), distributed across six runs (total duration of the experiment: 41 min). For the purposes of this study, we only considered blocks with facing and non-facing dyads, which were presented in three runs, each lasting 6.83 min. Each run consisted of 2 sequences of 16 blocks (4 per condition), yielding a total of 32 blocks. Blocks in the first sequence were presented in a random order, and blocks in the second sequence were presented in the counterbalanced order relative to the first one. Each run began with a 10 s warm-up block and ended with a 16 s cool-down block during which a black fixation cross was presented. The onset time of each block was jittered within a run (range of inter-block interval duration: 2–6 s; total inter-block time by runs: 128 s) using the optseq tool of Freesurfer^46^ to optimize jittering. Each block consisted of eight 550 ms stimuli of the same condition presented in a random order, separated by a 450 ms interval. Each stimulus appeared once in a block and twice in a run (one in each sequence). A black fixation cross was always present on the screen. Participants were instructed to fixate the cross throughout the experiment and to press a button with their right index finger when the cross turned red (cross changed color in 37% of the stimulation and fixation periods). This task was implemented to minimize eye movements and maintain vigilance in the scanner. During this session, participants also completed two runs of a standard functional localizer task (total duration: 10.54 min) adapted from the fLoc package^47^, to define, in individual participants, regions of interest (ROIs) responding to visual perception of bodies. Stimuli for this task consisted of 180 grayscale photographs of bodies (headless bodies and body parts), faces, places (houses and corridors), inanimate objects and scrambled objects (see Abassi and Papeo^2^ for details).

Dynamic stimuli were presented during an event-related fMRI design, including two stimulation runs with PLDs of single bodies and two stimulation runs with PLDs of facing and non-facing body dyads, randomly interleaved (total duration: 34 min 8 s). For the purposes of this study, we only considered trials with facing and non-facing dyads. Facing and non-facing dyads were presented randomly over two functional runs, each lasting 8 min 32 s. Each run consisted of 2 sequences separated by a 16 s interval. Each sequence was composed of 40 stimuli (2 s movies of facing and non-facing dyads), with an inter-stimulus interval (ISI) of 2, 4, of 6 s, each occurring with 1/3 probability. Events in the first sequence were presented in a random order, and events in the second sequence were presented in the counterbalanced order relative to the first one. Each stimulus was repeated twice in a sequence (original view and its flipped version), hence, 8 times across the experiment (4 times in each run). Each run began with a warm-up block (8 s) and ended with a cooldown block (16 s), during which a central fixation cross was presented. To minimize eye movements and maintain vigilance, participants were instructed to fixate the center of the screen and to press a button with their right index finger when the dots forming the point-light displays changed color (dots went from white to light pink in 2.5% of events across a run). During this session, participants also completed two standard functional localizer tasks (total duration: 21 min 32 s). In one task (motion-localizer task), stimuli were arrays of white dots on a black background moving in coherent motion, alternating with arrays of static dots. This task was used to localize the motion-responsive middle temporal visual area (MT/V5) (see^48^). In the other task (biological motion-localizer task), one condition involved PLDs (white dots on a black background) depicting the motion of a human body; the other condition involved scrambled-PLDs that retained local motion-information (the motion of individual dots) but presented dots in different location relative to the PLD condition, so that human body and motion were no longer recognizable (see^48^). With this task we localized the pSTS area responsive to biological motion, which is adjacent to and overlapping with pSTS areas showing effects of social interaction (see^3,4^). However, since neuronal populations with different functional specificities are present in the pSTS, we labeled our functionally-localized pSTS area as ‘BM-pSTS’ (biological-motion pSTS) to be explicit about how this ROI was defined.

In both experiments, stimuli were back-projected onto a translucent screen by a liquid crystal projector (frame rate: 60 Hz; screen resolution: 1024 × 768 pixels, screen size: 40 × 30 cm). Participants viewed the screen binocularly (7° of visual angle) through a mirror above the head coil. Stimulus presentation, response collection, and synchronization with the scanner were controlled with the Psychtoolbox^49–51^ through MATLAB (MathWorks, Natick, MA).

### Data acquisition

Imaging was conducted on a MAGNETOM Prisma 3T scanner (Siemens Healthcare). In both sessions (static and dynamic stimuli), T2*-weighted functional volumes were acquired using a gradient-echo-planar imaging (GRE-EPI) sequence (acquisition parameters: repetition time (TR) 2000 ms; echo time (TE) 30 ms, flip angle 80°; acquisition matrix 96 × 92; field of view (FOV) 210 × 201; 56 transverse slices; slice thickness 2.2 mm; no gap; multiband acceleration factor 2; phase encoding set to anterior/posterior direction). T1-weighted images were acquired with an MPRAGE sequence (TR/TE/TI 3000/3.7/1100 ms; flip angle 8°, acquisition matrix 320 × 280; FOV 256 × 224 mm; slice thickness 0.8 mm; 224 sagittal slices, GRAPPA accelerator factor 2). The acquisition of two field maps was performed at the beginning of each fMRI session (both for static and dynamic stimuli sessions). In the session with static stimuli, eight runs were acquired for a total of 1546 frames per participant, for the main experiment and the functional localizer task. In the session with dynamic stimuli, 6 runs were acquired for a total of 1374 images per participant, for the main experiment and the functional localizer task.

### Analyses

#### Preprocessing of B0 inhomogeneity mappings and functional images

fMRI data were treated *ex novo* using the optimized preprocessing pipeline fMRIPrep 22.0.2^52,53^, based on Nipype 1.8.5^54,55^. One fieldmap was used for each participant. A B_0_ nonuniformity map (or fieldmap) was estimated from the phase-drift maps measure with two consecutive GRE (gradient-recalled echo) acquisitions. The corresponding phase-maps were phase-unwrapped with prelude (FSL 6.0.5.1:57b01774). The T1-weighted (T1w) image was corrected for intensity non-uniformity (INU), and used as T1w-reference throughout the workflow. Brain extraction, surface reconstruction and brain tissue segmentation of cerebrospinal fluid (CSF), white-matter (WM) and gray-matter (GM) were performed on the brain-extracted T1w. Volume-based spatial normalization to MNI (Montreal Neurological Institute) standard space was finally performed. For each participant, for each of the functional runs, preprocessing steps included: head motion parameter estimation, slice-timing correction, realignment, and co-registration to the T1w reference. Nuisance covariates such as head-motion parameters, WM and CSF signals were removed, and images were normalized to MNI standard space. After preprocessing, each functional volume was smoothed by a 6mm full-width at half-maximum (FWMH) Gaussian kernel, using a custom-made MATLAB code in combination with SPM12^56^. Time series for each voxel were high-pass filtered (cutoff 128 s) to remove signal drift and low-frequency noise. Further details on preprocessing are reported as Supplementary material 4.

#### Whole-brain analysis

**Fig. 2:**
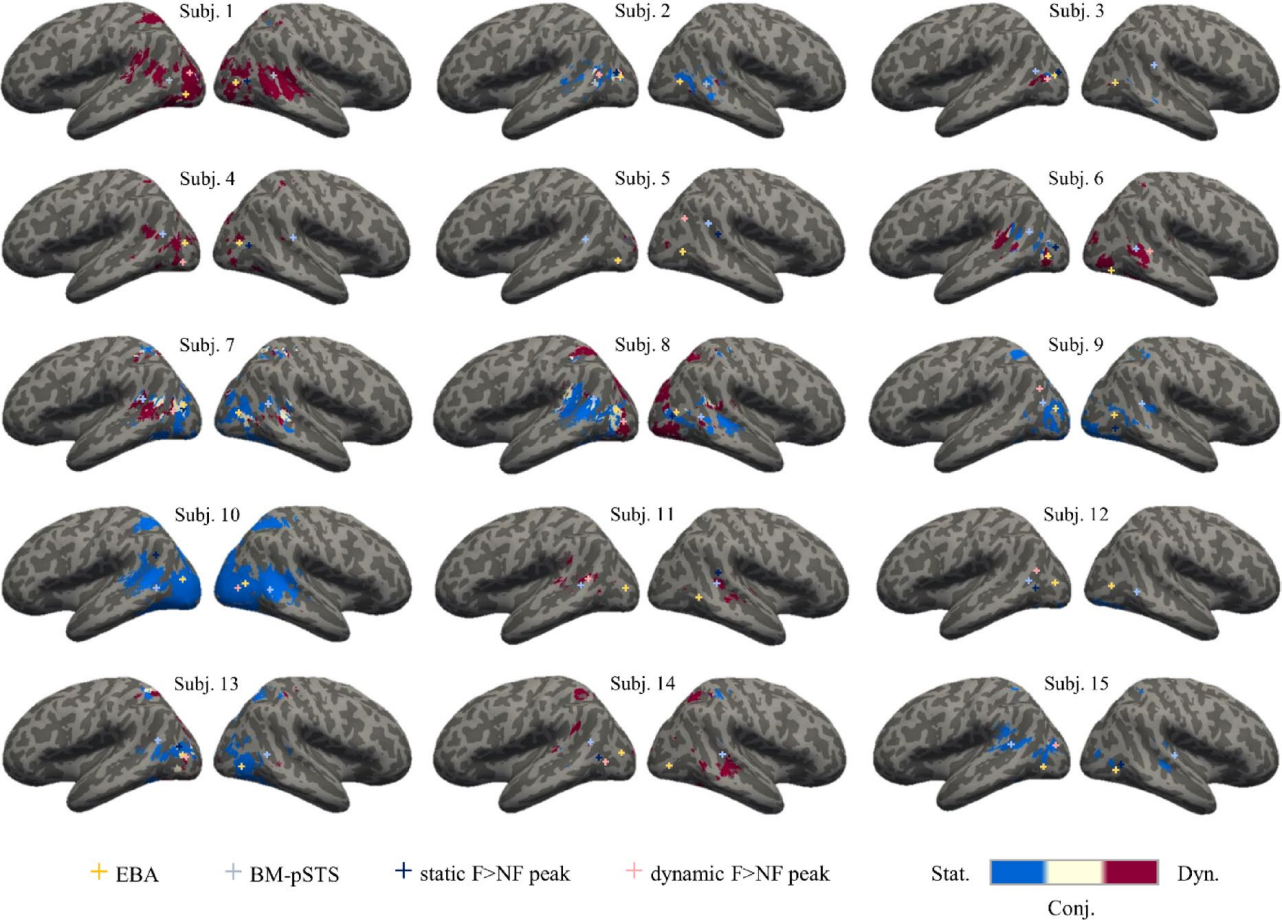
Individual maps for the conjunction (beige) of the facing > non-facing effect with static (blue) and dynamic (dark red) stimuli. Yellow crosses denote the individual’s EBA (from functional localizer data); light blue crosses denote the individual’s BM-pSTS (from functional localizer data); dark blue crosses denote the peak of the individual’s facing > non-facing effect for static stimuli; light pink crosses denote the peak of the facing > non-facing effect for dynamic stimuli. Group-level coordinates were used for defining the BM-pSTS of participant 6, 8 and 13 and the EBA of participant 2 since no reliable cluster was found near these regions.

For each participant, for each voxel, the BOLD signal was estimated in a general linear model (GLM) using SPM12, separately for the two datasets with static and dynamic stimuli. The GLM for the *static* dataset modeled two regressors for the two critical conditions—upright facing and non-facing dyads—, two regressors for the inverted facing and non-facing dyads conditions, and six regressors for movement correction parameters as nuisance covariates. For the *dynamic* dataset, the GLM included two regressors for the conditions with facing and non-facing dyads, and six regressors for movement correction parameters as nuisance covariates. We computed the contrasts facing > non-facing dyads and non-facing > facing dyads for static and dynamic stimuli. Statistical significance of second-level effects was determined using a voxelwise threshold of *p* < 0.001 with FDR-correction at the cluster level. In addition, subject-specific activity peaks for the facing > non-facing contrast in static and dynamic stimuli were gathered, within a custom-made bilateral mask encompassing EBA and pSTS, to examine the lateralization of these peaks and their proximity with the functionally localized EBA and BM-pSTS in each subject (Fig. 2).

#### Conjunction analysis

A conjunction analysis was carried out to identify the neural activity associated with the perception of facing (vs. non-facing) dyads irrespective of the stimulus modality (static or dynamic) and the activity tied to a specific modality. We considered the group-level maps obtained from the facing > non-facing contrasts for each set of stimuli (*p* < 0.001 at the voxel level, FDR corrected at the cluster level; Table 2), and assigned to each voxel a value of 1, 2 or 3, indicating respectively whether activity was above threshold for both the *static* and *dynamic* facing > non-facing contrast, for the dynamic contrast only, or for the static contrast only^57^. Individual conjunction maps were also plotted with an uncorrected threshold of *p* < 0.05 to examine subject-specific location of the conjunction (Fig. 2).

**Table 2:** Results of whole-brain analyses.

Condition	Contrasts	Hemisphere	Locations	Peak coordinates			*p*	Size
				x	y	z		
Static	Facing > Non-facing	Left	Middle occipitotemporal gyrus	− 55	− 80	14	0.021	48
Dynamic	Facing > Non− facing	Left	Middle occipitotemporal gyrus	− 51	− 69	10	< 0.001	431
			Superior parietal lobule	− 35	− 65	47	0.001	106
		Right	Middle occipitotemporal gyrus	50	− 52	10	0.008	63
	Non-facing > Facing	Left	Posterior occipital lobe	− 31	− 106	3	0.001	56

Activity peaks and statistics for the whole-brain contrasts facing > non-facing dyads and non-facing > facing dyads for static and dynamic stimuli.

*p* values are FDR-corrected at the cluster level (*p* = 0.001); Size corresponds to number of voxels in the cluster; x, y and z are the coordinates in the MNI space.

#### ROI definition and analyses

Using data from the functional localizer tasks, five regions of interest (ROIs) were identified in each participant: the extrastriate body areas (EBA), the motion-selective area in the middle temporal visual area (MT/V5), the pSTS region responsive to biological motion (BM-pSTS), the place-selective parahippocampal place area (PPA) and the early visual cortex (EVC). These ROIs were targeted based on previous studies showing social interaction selectivity in regions overlapping with EBA^1,2,11,15,17^, MT/V5^12,58^ and pSTS^3,4,7,28^. PPA and EVC were included as control areas, to test for the specificity of the effects in body- or motion-selective ROIs. To define the ROIs, first, in second-level analyses, we found the peaks of activity for the contrasts bodies > other objects for EBA, moving > static dots for MT/V5, PLDs of human motion > scrambled PLDs for BM-pSTS, places > other objects (faces, bodies, inanimate objects) for PPA, and all objects > baseline for EVC. Second, around these MNI-peak coordinates, for each ROI, we defined a sphere of 10-mm diameter (Marsbar Toolbox in SPM12^59,60^. All ROIs were defined bilaterally. Third, the spherical ROIs were used as masks to constrain the individual ROIs using the functional *t*-maps for the contrasts of interest of each subject. For each subject, voxels that were activated by given contrast (e.g., bodies > other objects) and that fell within the mask, were ranked based on *t* values; the 100 best voxels with a positive *t* value were included in the ROI. This process was performed separately for the two hemispheres. Overlapping voxels between two ROIs were removed (see supplementary Table S2).

For each participant, for each stimulus modality (static and dynamic), mean activity values (β-weights minus baseline) for facing and non-facing dyads were extracted from each left and right ROI, and analyzed in a 2 hemisphere × 5 ROI × 2 stimulus modality × 2 configuration repeated-measures ANOVA. Critical comparisons were performed with pairwise *t* tests (*p* < 0.05, two-tail). In addition to the frequentist ANOVA, Bayes Factors (BF_10_) were computed for each main effect and interaction to quantify the relative evidence for including stimulus modality as a factor in the model. For each comparison, we contrasted the model including the stimulus modality term with the corresponding model excluding it. The resulting Bayes Factors (BF_10_) reflect the probability of the data are under one model compared to the other. Interpretation of Bayes Factors followed conventional guidelines^61^.

## Results

### Effect of *facingness* for static and dynamic stimuli

We first examined the effect of body configuration (facing vs. non-facing) separately for each stimulus modality (static and dynamic). With static stimuli, the whole-brain contrast facing > non-facing revealed activity in a left-lateralized network centered in the lateral middle occipitotemporal gyrus, overlapping with the functionally-localized EBA (Table 2; Fig. 1b). The same contrast for dynamic stimuli revealed activity in a wider cluster encompassing the middle occipitotemporal gyrus bilaterally, and the left superior parietal lobule (Table 2; Fig. 1b.), showing that motion indeed increased the overall responsiveness of the network to social interactions. The contrast non-facing versus facing revealed activity in the left posterior occipital lobe for dynamic stimuli only (Table 2; unthresholded activity maps are provided in Supplementary Fig. S1).

The conjunction of the activation maps for the facing > non-facing contrast for static and dynamic stimuli revealed activity in the left middle occipitotemporal cortex, which overlapped with the functionally-localized EBA (Fig. 1c). At the individual-subject level, we observed that peaks were more often localized in the left hemisphere (number of subjects: static_left_ = 8; static_right_ = 7; dynamic_left_ = 13; dynamic_right_ = 2; see Fig. 2). Conjunction maps for individual subjects, with activity peaks for the static and dynamic contrast [facing > non-facing] and functionally-localized EBA and BM-pSTS, are plotted in Fig. 2. To extract these peaks for each participant we (1) computed the first-level contrasts [bodies > other objects] and [biological motion > scrambled motion]; (2) identified the clusters in the left and right hemisphere closest to the anatomical locations, based on probabilistic maps (i.e. meta-analysis from Neurosynth.org, search terms ‘occipitotemporal cortex’ and ‘pSTS’), of the EBA (i.e. lateral occipitotemporal cortex) and pSTS (i.e. posterior part of the sulcus), respectively (3) extracted the coordinates of the peak voxel in each cluster. MNI coordinates of EBA, BM-pSTS and peaks can be found in the supplementary Table S1.

### Effect of *facingness* for static and dynamic stimuli in motion- and body-perception ROIs

The ANOVA showed the main effects of hemisphere (mean_difference_ ± sd = 1.56 ± 1.46; *F*_1,14_ = 16.93, *p* = 0.001, *η*_*p*_^*2*^ = 0.55, Bayes Factor (BF_10_) = 8.11e + 10), ROI (*F*_4,56_ = 51.36, *p* < 0.001, *η*_*p*_^*2*^ = 0.79, BF_10_ = 6.85e + 25), stimulus modality (mean_difference_ ± sd = 3.46 ± 2.39; *F*_1,14_ = 31.46, *p* < 0.001, *η*_*p*_^*2*^ = 0.69, BF_10_ = 1.76e + 27) and configuration (mean_difference_ ± sd = 0.59 ± 0.84; *F*_1,14_ = 7.43, *p* = 0.016, *η*_*p*_^*2*^ = 0.35, BF_10_ = 4.75e + 10), indicating that overall activity was stronger in the left (vs. right) hemisphere, differed across ROIs, and was stronger for dynamic (vs. static stimuli) and for facing (vs. non-facing) stimuli.

A significant interaction between ROI and stimulus modality (*F*_4,56_ = 6.63, *p* < 0.001, *η*_*p*_^*2*^ = 0.32, BF_10_ = 510.28) showed that all ROIs but the EBA responded more strongly to dynamic than static stimuli (EBA: mean_difference_ ± sd = 2.55 ± 4.92, CI [− 0.18;5.27], *t*(*14*) = 2.01, *p* = 0.065, *d* = 0.52; BM-pSTS: 2.85 ± 2.29, CI [1.58;4.12], *t*(*14*) = 4.82, *p* < 0.001, *d* = 1.24; MTV5: 4.50 ± 3.78, CI [2.41;6.59], *t*(*14*) = 4.61, *p* < 0.001, *d* = 1.19; PPA: 1.64 ± 1.16, CI [0.99;2.29], *t*(*14*) = 5.47, *p* < 0.001, *d* = 1.41; EVC: 5.77 ± 2.86, CI [4.19;7.36], *t*(*14*) = 7.81, *p* < 0.001, *d* = 2.02), but the difference was stronger in MTV5 as compared to BM-pSTS (1.65 ± 2.73, CI [0.13;3.16], *t*(*14*) = 2.33, *p* = 0.035, *d* = 0.60) and PPA (2.85 ± 3.89, CI [0.70;5.01], *t*(*14*) = 2.84, *p* = 0.01 , *d* = 0.73), and in EVC as compared to BM-pSTS (2.92 ± 2.99, CI [1.26;4.58], *t*(*14*) = 3.78, *p* = 0.002, *d* = 0.98) and to PPA (4.13 ± 2.93, CI [2.51;5.75], *t*(*14*) = 5.46, *p* < 0.001, *d* = 1.41). The Stimulus Modality did not interact with any other factor including Hemisphere.

Instead, we found significant interactions between Hemisphere and ROI (*F*_4,56_ = 3.18, *p* = 0.020, *η*_*p*_^*2*^ = 0.19, BF_10_ = 4.58e + 27), Hemisphere and Configuration (*F*_1,14_ = 17.21, *p* < 0.001, *η*_*p*_^*2*^ = 0.55, BF_10_ = 7.21e + 10) and ROI and Configuration (F_4,56_ = 10.32, *p* < 0.001, *η*_*p*_^*2*^ = 0.42, BF_10_ = 1.33e + 26). These interactions were qualified by a significant three-way interaction between Hemisphere, ROI and Configuration (F_1,14_ = 4.94, *p* = 0.002, *η*_*p*_^*2*^ = 0.26, BF_10_ = 1.06e + 28). All other interactions were not significant (Hemisphere x Stimulus modality: F_1,14_ = 0.02, p = 0.885 , *η*_*p*_^*2*^ < 0.01, BF_10_ = 0.12; Stimulus modality x Configuration: F_1,14_ = 0.18, *p* = 0.674, *η*_*p*_^*2*^ = 0.01, BF_10_ = 0.13; Hemisphere x ROI x Stimulus modality: F_4,56_ = 1.07, *p* = 0.379 , *η*_*p*_^*2*^ = 0.07, BF_10_ = 0.04; Hemisphere × Stimulus modality × Configuration: F_1,14_ = 0.06, *p* = 0.805, *η*_*p*_^*2*^ < 0.01, BF_10_ = 0.18; ROI × Stimulus Modality × Configuration: F_4,56_ = 0.11, *p* = 0.980, *η*_*p*_^*2*^ < 0.01, BF_10_ = 0.02; ROI × Stimulus modality × Configuration × Hemisphere: F_4;56_ = 1.49, *p* = 0.219, *η*_*p*_^*2*^ = 0.10, BF_10_ = 0.06).

First, the above results showed that whether stimuli were static or dynamic did not seem to affect the lateralization of social interaction selectivity. In line with this, the BF_10_ (Bayes factors) reported above showed decisive evidence in favor of the three-way interaction between ROI, hemisphere and stimulus modality, but also substantial to very strong evidence for the lack of interaction of stimulus modality with configuration or other factors.

Second, to investigate the three-way interaction further, we considered to what extent the social interaction selectivity differed between the homologous left and right ROIs. As shown in Fig. 1d, the *facing* > *non-facing* effect was especially stronger in the left (vs. right) EBA, BM-pSTS and MT, with no difference between static and dynamic stimulus-conditions. Statistics (i.e., Hemisphere x Configuration ANOVAs run for each ROI separately) confirmed this observation.

#### EBA

The ANOVA revealed significant main effects of hemisphere (mean_difference_ ± sd = 2.34 ± 3.71; *F*_1,14_ = 6.00, *p* = 0.028, *η*_*p*_^*2*^ = 0.23) and configuration (mean_difference_ ± sd = 1.26 ± 1.17; *F*_1,14_ = 17.29, *p* = 0.001, *η*_*p*_^*2*^ = 0.46), and a significant interaction between hemisphere and configuration (*F*_1,14_ = 31.35, *p* < 0.001, *η*_*p*_^*2*^ = 0.43), showing that the difference between facing and non-facing dyads was significant in both left and right ROIs (left: mean_difference_ ± sd = 1.83 ± 1.35, CI [1.09;2.58], *t*(*14*) = 5.26, *p* < 0.001, *d* = 1.36; right: 0.69 ± 1.12, CI [0.07;1.31], *t*(*14*) = 2.39, *p* = 0.031, *d* = 0.62), but it was stronger in the left side.

#### MT/V5

The ANOVA revealed a main effect of configuration (mean_difference_ ± sd = 0.76 ± 1.00, *F*_1,14_ = 8.72, *p* = 0.010, *η*_*p*_^*2*^ = 0.43), no effect of hemisphere (mean_difference_ ± sd = 0.97 ± 4.20, *F*_1,14_ = 0.80, *p* = 0.385, *η*_*p*_^*2*^ = 0.07), and a significant interaction between hemisphere and configuration (*F*_1,14_ = 6.78, *p* = 0.021, *η*_*p*_^*2*^ = 0.23). Like in the EBA, in the MT/V5, the facing > non-facing effect was significant in both sides, but stronger in the left (left: mean_difference_ ± sd = 0.99 ± 1.24, CI [0.31,1.68], *t*(*14*) = 3.11, *p* = 0.008, *d* = 0.80; right: mean_difference_ ± sd = 0.53 ± 0.84, CI [0.07,0.99], *t*(*14*) = 2.45, *p* = 0.028, *d* = 0.63).

#### BM-pSTS

Like MT/V5, BM-pSTS showed a main effect of configuration (mean_difference_ ± sd = 0.98 ± 0.81, *F*_1,14_ = 22.03, *p* < 0.001, *η*_*p*_^*2*^ = 0.83), no main effect of hemisphere (mean_difference_ ± sd = 1.50 ± 2.74, *F*_1,14_ = 4.51, *p* = 0.052, *η*_*p*_^*2*^ = 0.50), and an significant interaction (*F*_1,14_ = 22.66, *p* < 0.001, *η*_*p*_^*2*^ = 0.72), showing that the facing > non-facing effect was significant in both sides but stronger in the left (left: mean_difference_ ± sd = 1.50 ± 1.12, CI [0.88;2.13], *t*(*14*) = 5.18, *p* < 0.001, *d* = 1.33; right: mean_difference_ ± sd = 0.45 ± 0.64, CI [0.10;0.80], *t*(*14*) = 2.74, *p* = 0.016, *d* = 0.71).

#### PPA and EVC

We found a main effect of hemisphere in EVC (left > right; mean_difference_ ± sd = 3.16 ± 1.70, *F*_1,14_ = 51.84, *p* < 0.001, *η*_*p*_^*2*^ = 0.86). No other effect was significant in EVC (configuration: mean_difference_ ± sd = 0.01 ± 1.68, *F*_1,14_ = 0.01, *p* = 0.907, *η*_*p*_^*2*^ < 0.01; interaction: *F*_1,14_ = 0.10, *p* = 0.752, *η*_*p*_^*2*^ < 0.01). No effect was significant in the PPA (hemisphere: *F*_1,14_ = 3.53, *p* = 0.08, *η*_*p*_^*2*^ = 0.97; configuration: *F*_1,14_ < 0.01, *p* = 0.999, *η*_*p*_^*2*^ < 0.01; interaction: *F*_1,14_ = 1.27, *p* = 0.278, *η*_*p*_^*2*^ = 0.86).

## Discussion

There is growing evidence of the existence of a specialized network for processing of third-party social interactions, rooted in visual perception and in visual cortex ^6,7,14,41,62^, and unfolding along a lateral occipitotemporal pathway, from V1 to EBA, MT/V5 and BM-pSTS. Here, we highlighted this network by contrasting the neural response to viewing interacting vs. non-interacting individuals. Moreover, we tested whether *social interaction selectivity* —i.e., the stronger response to interacting versus non-interacting bodies—was affected by the stimulus modality; particularly, we asked whether motion is a necessary stimulus property, or the mere perception of two bodies close together and oriented towards each other is sufficient to elicit social interaction selectivity (see for discussions on this issue^12,39–41,61^). Results showed that (i) despite the overall greater activity elicited by dynamic stimuli, no reliable difference was observed between dynamic and static stimuli in the degree of social interaction selectivity in the network encompassing the EBA and pSTS; (ii) social interaction selectivity was found even though stimuli did not depict meaningful, fully-fledged, easy to identify, social interactions; and (iii) it was stronger in the left than in the right hemisphere, for both static and dynamic stimuli.

The present study is the first to compare the effect of social interaction selectivity between static and dynamic representations of social interactions, for minimal social scenes featuring just two people close together and face-to-face. This comparison established that a pathway of areas in the lateral visual cortex is tuned to visual scenes that carry basic, reliable cues of social interaction, such as *facingness* between two nearby individuals^6,63–65^, without further perceptual and non-perceptual cues to aid social interaction recognition and specify the content of interaction. Among other cues, motion has been proposed to play a key role in social interaction perception. One argument for this claim is that, in real world, social interactions typically are dynamic events, and therefore motion cues are part of the routine processing and recognition of social interactions. In effect, regions along the visual pathway that respond to perception of social interactions are also implicated in processing motion, in extracting action/movement representation combining body-posture and motion information (through connections between EBA and pSTS^3^), and respond more strongly to moving than static faces and bodies^41^.

The most direct evidence in favor of a key role of motion in social interaction perception was reported in Landsiedel et al.^12^, who found selectivity to social interactions in EBA and pSTS, only for dynamic representations of social interaction (video-clips), but not for their static counterpart (photos of the most informative frame of a clip). While both Landsiedel et al. and the present study compared effects between static and dynamic stimuli, a critical difference is that, here, we reduced social interaction to a ‘critical minimum’ (i.e., *facingness* with spatial proximity); instead, Landsiedel et al. used naturalistic every-day social scenes, in which the interaction was conveyed by a rich set of cues (visuo-spatial such as distance, body posture and orientation, and contextual such as objects, place, clothing), which did not necessarily include the ‘critical minimum’ (e.g., in Fig. 1 of Landsiedel et al. two people interact in a street without ever being face-to-face). We can confidently exclude that, in our study, the lack of an effect of motion on the selectivity to social interaction reflects possible limitations of the study. At least, despite the small sample size, our dataset had sufficient statistical power to detect small-to-medium effects (see “Participants” section); and, Bayesian statistics supported the lack of interaction between stimulus modality and configuration (see “Results” section). Thus, the present results showed a critical effect of *facingness* in triggering the selectivity to social interaction, which generalized across stimuli that were very visually different: static human bodies and animated point-light-displays. In this light, a possible synthesis of the available results is that the visual system is tuned to the perception of bodies that carries prototypical cues of social interaction such as *facingness*; the presence of such cues is sufficient to trigger the social-interaction perception pathway up to the pSTS, even in the absence of motion information. This empirical fact is consistent with the astonishing human ability to detect social interactions^5,8^, categorize them^66^, judge their coherence^67,68^, and assign roles (agent/patient) to the event participants^69–71^, upon brief presentation (even 33 ms) of static visual images, provided that those images carry prototypical cues of interaction such as facingness, spatial proximity and/or contact.

While *facingness* has been extensively investigated as a basic social interaction cue^6,10,72,73^, it is likely not the only ‘social primitive’^14^. Motion, particularly self-propelled motion, remains a typical and important component of social interaction, however, it might be too unspecific to be a reliable ‘social primitive’, as it is a property of biological agents^74^, whether they do or do not interact with each other. An exhaustive list of ‘social primitives’ will advance our understanding of how representations of social interactions can be constructed in the human mind/brain as well as in artificial systems^75^.

Results from whole-brain, conjunction, and region of interest analysis also showed stronger social interaction selectivity in the left than right visual areas, for both static and dynamic stimuli. This left-lateralization of the effect is at variance with the broad literature suggesting a prominent role of the right hemisphere in social vision, i.e., the visual perception of social stimuli such as faces, bodies and biological motion^76–81^ and social cognition (e.g., in theory-of-mind tasks^82,83^).

Focusing on perception of social interactions, while the earliest reports emphasized a selective involvement of right visual areas^4,7^, strong claims for right-lateralized effects have vanished in more recent studies^1,2,11,12,17,37^ (see Table 1). One possibility is that perception of social interaction is less right-lateralized than initially thought, or not at all. Here, however, we found evidence for a left-lateralization of the effects. This circumstance is in line with recent research showing that disrupting left EBA activity with transcranial magnetic stimulation (TMS) alters visual discrimination of face-to-face (vs. back-to-back) bodies^20^. Stimuli in that TMS study^20^ were analogous the static set used here. Thus, one hypothesis is that left-lateralized selectivity is associated with the perception of stimuli that carry prototypical cues of social interaction (i.e., *facingness* and spatial proximity), while right activity is triggered by richer stimuli that specify the content of the interaction. Encouraging this thinking, our review of the literature (Table 1) suggests that studies reporting right-lateralized or bilateral effects in visual areas involved meaningful, easy to identify, dyadic social interactions, or naturalistic depictions of social scenes, while more basic visual representations of social interactions mainly triggered left lateralized effects.

This circumstance suggests the intriguing idea of a division of labor within the social-interaction perception system, where left areas encode the semantic (or *thematic*) structure of the interaction based on spatial and postural relations that define the number of participants and their role in the event, while right areas encode information relevant for narrower event-category distinctions (e.g., helping vs. hindering^4,7^), attribution of goals and intentions and other social-semantic contents. Our stimuli, reducing social interactions to the ‘critical minimum’, would drive activity in left areas, while missing the additional information that specifies event category, goals and intentions, supporting *mind reading* and other social-cognitive operations.

The results discussed here support the existence of a pathway that, with a sequence of hierarchically-organized stages, moves from processing visual features of social interactions to processing higher-level properties^3,14,41^. Researchers in the field have an exciting road ahead of them to determine how many functionally different regions exist along this pathway, what their functions are, and how these functions are integrated to represent social interaction. Moreover, all the brain areas targeted in this study—and in this research field more generally—are well known for functions other than social-interaction perception. For example, EBA is known for its role in body/body part perception and MT is known for its role in motion perception. Since the very same bodies and body motion are involved in facing and non-facing stimuli (see “Stimuli” section), what accounts for the effects of *facingness* in these areas? The available results do not provide an answer to this question, but they clearly show that current knowledge does not exhaust the functions of those brain areas that have been studied for several decades now. Another challenge will be to explain the inter-hemispheric dynamics that integrate information from the right and left regions of this pathway, recognizing that there is a specific place for the left hemisphere in the social brain.

## Electronic supplementary material

Below is the link to the electronic supplementary material.

Supplementary Material 1

## Data Availability

Stimuli, analysis codes and supplementary materials associated with this article can be found online at https://osf.io/mbzfs/?view_only=33c30f08f9b84a8692bdb59fd3945497.
